# Improved packing of protein side chains with parallel ant colonies

**DOI:** 10.1186/1471-2105-15-S12-S5

**Published:** 2014-11-06

**Authors:** Lijun Quan, Qiang Lü, Haiou Li, Xiaoyan Xia, Hongjie Wu

**Affiliations:** 1 School of Computer Science and Technology, Soochow University, Suzhou, 215006, China; 2 Jiangsu Provincial Key Lab for Information Processing Technologies, Suzhou, 215006, China; 3 School of Electronic and Information Engineering, Suzhou University of Science and Technology, Suzhou, 215006, China

**Keywords:** protein, side chains, pack, ACO, parallel

## Abstract

**Introduction:**

The accurate packing of protein side chains is important for many computational biology problems, such as ab initio protein structure prediction, homology modelling, and protein design and ligand docking applications. Many of existing solutions are modelled as a computational optimisation problem. As well as the design of search algorithms, most solutions suffer from an inaccurate energy function for judging whether a prediction is good or bad. Even if the search has found the lowest energy, there is no certainty of obtaining the protein structures with correct side chains.

**Methods:**

We present a side-chain modelling method, pacoPacker, which uses a parallel ant colony optimisation strategy based on sharing a single pheromone matrix. This parallel approach combines different sources of energy functions and generates protein side-chain conformations with the lowest energies jointly determined by the various energy functions. We further optimised the selected rotamers to construct subrotamer by rotamer minimisation, which reasonably improved the discreteness of the rotamer library.

**Results:**

We focused on improving the accuracy of side-chain conformation prediction. For a testing set of 442 proteins, 87.19% of $X_{1}$ and 77.11% of $X_{12}$ angles were predicted correctly within 40° of the X-ray positions. We compared the accuracy of pacoPacker with state-of-the-art methods, such as CIS-RR and SCWRL4. We analysed the results from different perspectives, in terms of protein chain and individual residues. In this comprehensive benchmark testing, 51.5% of proteins within a length of 400 amino acids predicted by pacoPacker were superior to the results of CIS-RR and SCWRL4 simultaneously. Finally, we also showed the advantage of using the subrotamers strategy. All results confirmed that our parallel approach is competitive to state-of-the-art solutions for packing side chains.

**Conclusions:**

This parallel approach combines various sources of searching intelligence and energy functions to pack protein side chains. It provides a frame-work for combining different inaccuracy/usefulness objective functions by designing parallel heuristic search algorithms.

## Introduction

The accurate packing of side chains plays a very important role in modelling protein structures. In ab initio structure prediction, the goal is to choose a rotamer for each position so that the molecule is close to the natural structure. In homology modelling, the goal is to predict the structure of a protein that is homologous to another of a known structure [1,2]. In protein design, the goal is to find an amino acids sequence that will fold into a particular backbone [3]. In flexible ligand docking, the goal is to display a structural change ranging from large movements of entire domains to small side-chain rearrangements in the binding site [4-6]. Based on Anfinsen's hypothesis [7], the problem of packing side chains is usually mapped into a combinatorial optimisation problem and can be solved in a number of ways. However, a fixed backbone, an energy function and a possible rotamer set are always foundations of this widely studied formulation. All the current existing algorithms for the side-chain problem can be divided into two categories, heuristic and deterministic.

The side-chain problems have been proven as non-deterministic polynomial-time hard (NP-hard) [8-10]. Even when an approximate solution is sought within *O*(*cnR*) from the optimum, where *c *is a constant, *n *is the number of residues and *R *is the average number of rotamers per residue [11,12], the packing side chains cannot be solved successfully. Computational complexity analysis suggests that any global optimisation algorithms for this problem may, in the worst case, run in exponential time [11]. When they converge, dead-end elimination (DEE) algorithms [13,14] are designed to find the global minimum energy. Heuristics are not guaranteed to find a global minimum, but they almost always find a low-energy conformation in a reasonable time [15]. Therefore, heuristic algorithms become a natural choice for tackling the side-chain modelling problem. Traditionally, all heuristic approaches solve such side-chain problems as a single-objective optimisation Problem (SOP), using Monte Carlo (MC) [16], Ant Colony (AC) [17], and Simulated Annealing (SA) [18]. Some of the heuristic methods combine multiple strategies, such as a combination of DEE and the A^* ^algorithm [19], and combination of SA and MC [20-22]. The common feature of these heuristic approaches is that they all use an optimisation based on a single objective function.

Another method for solving the side-chain problem was by using the theory of decomposing the underlining residue relationship. One such method is SCWRL [23-25,15], which is widely used because of its speed, accuracy and ease of use. SCWRL3 decomposes original residue graphs to connected subgraphs, which cannot be disconnected by the removal of a single vertex. They find the global minimal energy conformation for the residues in these subgraphs [25]. The authors who proposed the SCWRL methods also observed that residues with a single rotamer or a single neighbour can be eliminated from the residue graph. Then SCWRL4 [15] transfers the original residue graphs to a tree for speeding up the solver. However, in the tightly packed environments of protein interiors, these methods will inherently lead to atomic clashes and hinder the prediction accuracy. Therefore, a new method, CIS-RR, performs clash detection-guided iterative searches (CIS) of side-chain rotamers whilst continuously optimising side-chain conformations using a conjugate gradients method [26].

In general, methods for predicting side chains seem to be limited not by the quality of search algorithms, but also by the quality of the energy functions employed [23]. An energy function typically consists of a combination of weighted energy terms. It is not hard to find different approaches, which develope distinctive kinds of energy functions. For example, SCWRL3 use an energy function based on logarithmic probabilities of rotamers and a simple repulsive steric energy term [25]. However, SCWRL4 also uses a short-range, soft van der Waals interaction potential between atoms rather than the linear repulsive-only function used in SCWRL3, as well as an anisotropic hydrogen bond function similar to that used in Rosetta [15,27]. The energy function of CIS-RR is also a modified the energy function of SCWRL3. The first improvement is to add attractive energy and weights to the van der Waals potential. The second improvement is to penalise the drifting of side chain dihedral angles away from the nearest rotamer library values for the original rotamer term. The existence of different energy functions implies that all energy functions are inaccurate in a universal sense (inaccuracy), but each of them is very useful in some specific sense (usefulness). This hypothesis is referred to as the inaccuracy/usefulness property [28]. The approaches based on SOP all use a single inaccuracy energy function to model side chains, so the results are sometimes inaccurate in a quantitative sense for discriminating native or near-native conformations.

In this study, a novel approach is proposed to assemble the usefulness and decrease the inaccuracy of different energy functions. We believe that it is more reasonable to model packing side chains as a multi-objective optimisation problem (MOP). Different energy functions should be combined to the best possible extent. As this idea has been successfully applied to de novo prediction of protein backbone [28,29], we also used parallel ant colony optimisation based on SHOP (SHaring One Pheromone matrix) [30]. Our parallel strategy is not for speeding up the predictor, but can be used to hybridise the usefulness of different energy functions. All energy functions can be adopted by an individual colony. In this way, we can avoid the sensitivity of the optimised parameters of energy functions, so we expect to obtain better generality of our predictor. This parallel strategy has been validated experimentally.

## Methods

We propose a novel parallel ant colony optimisation (ACO) metaheuristic frame-work for packing protein side chains by single-heuristic multi-objective algorithms (SHMO) to reduce the inaccuracy of a single energy. We denote a heuristic algorithm by *h *and different energy functions by *ε *= *{E*_1 _, . . . , *E_k _}*, where the number of threads amount to *k*. This type of algorithm is generally denoted by $\prod_{h}\left(E_{i}|\text{Θ}\right)$ where Θ refers to the control parameters in terms of heuristic search algorithms and can usually be tuned empirically before starting, or adaptively during the algorithm [28]. In the pacoPacker algorithm, *h *adopts ACO, and Θ contains two variables, private and public. To be more specific, all ant colonies share one common pheromone matrix *T *as a public variable, and each ant colony has a private variable including heuristic matrix *H_i _*and two other parameters, *α_i _*and *β_i _*. *A *= *{α*_1 _, . . . , *α_k _}*, determines the importance of the pheromone and *B *= *{β*_1 _, . . . , *β_k _}*, determines the importance of the heuristic matrix *H *= *{H*_1 _, . . . , *H_k _}*. This paper's method can be described as $\prod_{AC}\left(E_{i}|\alpha_{i},\beta_{i},H_{i},T\right)$. The Rosetta3.4 platform [31] is quite mature and supports the object-oriented paradigm, therefore pacoPacker uses Rosetta3.4 for building rotamer libraries, constructing interaction graphs, and scoring structures. Using Rosetta3.4 and OpenMP [32], our scheme is easy to implement.

### Search space

For an amino-acid sequence *t *with *n *length of residues, its side chains are packed with the lowest free energy. Let the rotamer library for *t *be *R *= *{R*_1 _, . . . , *R_n_}*, where the rotamer set is $R_{i}=\left\{r_{1},...,r_{m_{i}}\right\}$ for each residue *i *in *t*, the number of rotamers belonging to *R_i _*amount to *m_i_*, and different rotamer sets have a different quantity of rotamers. Rotamers were read from Dunbrack backbone dependent rotamer library (2010 version), such that frequencies and dihedral angles varied with the backbone dihedral angles *Φ *and *ψ *[33].

### Energy function

We adopted the same energy functions used by Rosetta. These scores are combinations of different weights and energy items, such as residue-environment and residue-residue interactions, secondary structure packing, chain density and excluded volume [28]. It does not matter which function is more accurate as all the energy functions share the inaccuracy/usefulness property. The Rosetta energy functions are adopted here to illustrate the implementation of our parallel approach. We forked eight threads to run separately using different energy functions, which rule out any side-chain-independent energy terms. Different threads have different private variables, which are listed in Table 1. Table 1 shows the weight of each score term on different score functions. Each score term is represented by letter (A, B, etc.), which correspond to Table 2.

**Table 1:** Score function and ACO parameters.

Thread ID	Score function	Score terms																	*α*	*β*
				
		A	B	C	D	E	F	G	H	I	G	K	L	M	N	O	P	Q		
1	standard	0.8	0.44	0.65	0.004	0.49	0.56	1.17	1.17	1.17	1.1	0.5	2	5	5	1	0	0	3	1
2	score12	0.8	0.44	0.65	0.004	0.49	0.56	1.17	0.585	1.17	1.1	1	1	1	1	1	0	0	1	1
3	score12 full	0.8	0.44	0.65	0.004	0.49	0.56	1.17	0.585	1.17	1.1	0.5	2	5	5	1	0	0	1	2
4	score12minpack	0.8	0.44	0.65	0.004	0.49	0.56	1.17	0.585	1.17	1	1	1	1	1	1	0	0	1	3
5	score13	0.6921	0.1754	0.5253	-0.00764	0.53	0.63	1.322	0.336	2	1.883	0.5	2	5	5	1	0.571	0	2	1
7	score13	0.6921	0.1754	0.5253	-0.00764	0.53	0.63	1.322	0.336	2	1.883	0.5	2	5	5	1	0.571	0	1	1
8	pack no hb env dep	0.8	0.1	0.65	0.004	0.49	0.56	1.17	1.17	1.17	3.1	1	1	1	1	1	0	1	3	1
6	RosettaHoles score	The RosettaHoles scores are based on packing information about a cavity ball and the local region surrounding it, most importantly the contact surface area of atoms surrounding the cavity with respect to a sequence of probe radii.																	1	2

**Table 2:** Score terms.

Score term	Label	Description
fa_atr	A	lennard-jones attractive
fa_rep	B	lennard-jones repulsive
fa_sol	C	lazaridis-jarplus solvation energy
fa_intra_rep	D	lennard-jones repulsive between atoms in the same residue
fa_pair	E	pairwise electrostatics term derived from statistics on the pdb database
fa_dun	F	internal energy of sidechain rotamers as derived from Dunbrack's statistics
hbond_lr_bb	G	long range (beta or loop) backbone-backbone hydrogen bonds
hbond_sr_bb	H	short range (helix) backbone-backbone hbonds
hbond_bb_sc	I	sidechain-backbone hydrogen bond energy
hbond_sc	J	sidechain-sidechain hydrogen bond energy
dslf_ss_dst	K	distance score in current disulfide
dslf_cs_ang	L	csangles score in current disulfide
dslf_ss_dih	M	dihedral score in current disulfide
dslf_ca_dih	N	*Cα *dihedral score in current disulfide
pro_close	O	proline ring closure energy
envsmooth	P	Statistically derived fullatom environment potential
atom_pair_constraint	Q	Harmonic constraints between atoms involved in Watson-Crick base pairs specified by the user in the params file

### Implementation of the algorithm

**Figure 1:**
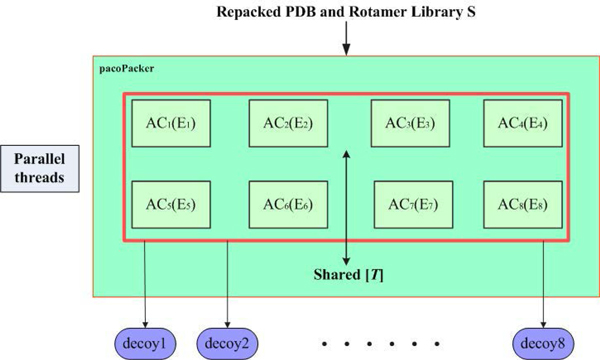
**pacoPacker schematic flowchart**.

Eight parallel threads were created in our SHMO implementation. Figure 1 depicts the design of pacoPacker. Using a protein backbone as the input of pacoPacker, the rotamer library is generated based on the target sequence by using the Rosetta platform. The outputs are proteins with side chains predicted by ant colonies. From the information shown in Figure 1, eight different ant colonies share a single common pheromone matrix *T *to exchange their search experience asynchronously. Each colony is directed by its own energy functions, which both co-evolve towards a better state.

Next, we will focus on a single ant colony to pack side chains. Construction by an ant colony is described as follows:

1. Conduct side chains based on the selection equation for each ant.

2. Perform the local search on each odd-numbered iteration ant.

3. Update global best ant *s_gb_*with iteration best ant *s_ib _*if *E*(*s_ib_*) is lower.

4. Update the pheromone matrix *T *based on *s_gb_*.

5. If the termination criterion is met, let's return to *s_gb_*, or repeat steps 1 to 5.

In this workflow, each colony terminates when one of the following criteria is met: the colony runs for a specified number of iterations; and there is no energy improvement during the last several iterations. Two important equations, the selection equation and the update pheromone matrix equation are explained below.

Each ant conducts the conformation by assembling rotamers from *R*. The ant picks up a rotamer *r_j _*from the rotamer set *R_i _∈ R *for residue *i*. For *g^th ^*thread, the rotamer selection is determined by the current heuristic and historical knowledge, described by the following selection equation (Equation 1):

(1) $$r_{j}^{*}=\left\{\begin{array}{cc} {\text{max}}_{r_{j}\in R_{i}}{\left[\tau_{ij}\right]}^{\alpha_{g}}{\left[\eta_{ij}\right]}^{\beta_{g}}, & \text{if}\;q<q_{0};\\ \text{randomly}\;\text{pick}\;\text{up}\;r_{j}\;\text{from}\;R_{i}, & \text{otherwise}\text{.} \end{array}\right)$$

Where *τ_ij _*is defined later in Equation 3, which denotes the useful experience accumulated by previous searches, *η_ij _*denotes the heuristic value. Let the heuristic matrix be: $H_{g}=\prod_{i\in n,j\in m_{i}}\eta_{ij}$, where *η_ij _*is the energy difference induced by residue *i *picking up rotamer *r_j _*, which is standardised according to Equation 2.

(2) $$\eta_{ij}=\frac{\pi }{2}-\text{arctan}\;\text{Δ}E.$$

*q*_0 _tunes the bias between the two selection policies. A random probability *q *will be generated when a rotamer is needed. Once the rotamer is picked, $r_{j}^{*}$ is inserted into the protein backbone from the position of residue *i*.

The second formula updates the pheromone matrix *T *after all the ants have finished their work in an iteration. Let the pheromone matrix be: $T=\prod_{i\in n,j\subseteq m_{i}}\tau_{ij}$, where *τ_ij _*is the pheromone value accumulated by residue *i *packing rotamer *r_j_*. For each *r_j _*of residue *i *in *s_gb_*, the value is updated using Equation 3.

(3) $$\tau_{ij}=\left(1-\rho \right)\tau_{ij}+\rho \text{Δ}\tau_{ij}.$$

Where ρ∈[0,1) is the pheromone evaporation factor, and Δ*τ_ij _*is calculated by a quality function which converts the energy value to a certain amount of pheromone. We describe this situation in Equation 4.

(4) $$\text{Δ}\tau_{ij}=\left\{\begin{array}{cc} \frac{\pi }{2}-\text{arctan}\frac{E\left(s_{gb}\right)}{n}, & \text{if}\;r_{j}\;\text{of}\;\text{residue}\;i\in s_{gb};\\ \tau_{ij}, & \text{otherwise}\text{.} \end{array}\right)$$

Our SHMO scheme is simple with the help of OpenMP. The pheromone matrix is extracted from AC, and multiple colonies are run as parallel threads with private variables in each colony to co-evolve with the common pheromone matrix.

### Rotamer minimization

**Figure 2:**
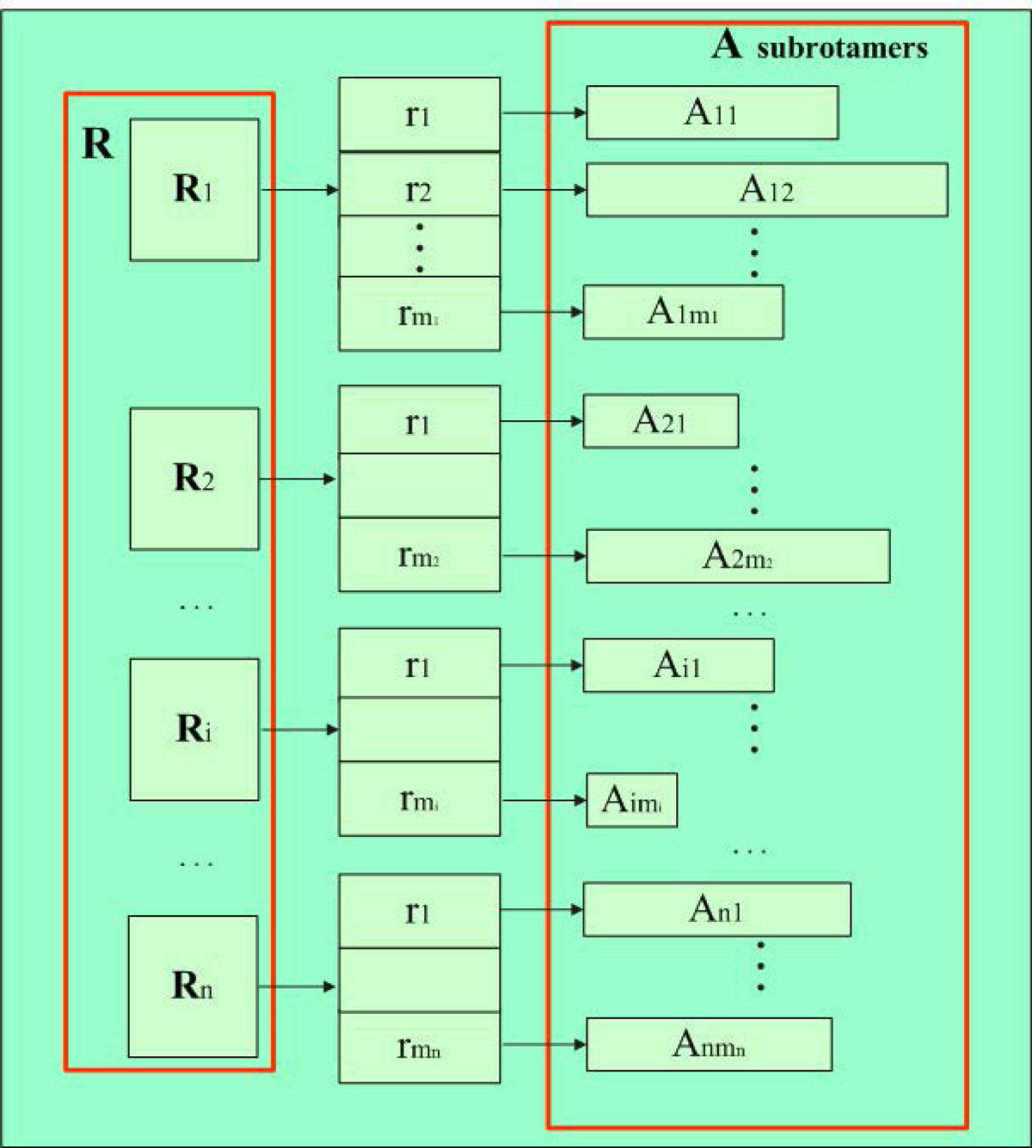
**Data structures of rotamers and subrotamers**.

Rotamer minimisation was implemented in two ways. First, the pacoPacker runs on each normal rotamer as it is placed; after that, the pacoPacker runs a global minimisation on the side chains at all the packable positions. We will not provide much detail about this method, as the Rosetta3.4 mechanism was adopted to achieve it. Second, pacoPacker runs a gradient minimisation on each rotamer as it is placed and keeps the minimised rotamers. To use this second method, we devised a new data structure to remember minimised rotamers (Figure 2). If there are *M *= *m*_1 _+ *m*_2 _+*· · ·*+*m_n _*rotamers, and each normal rotamer has its own alternative obtained by minimising itself, they are called subrotamers. We describe the set of subrotamers for *r_j _*from *R_i _*as *A_ij _*, which can be calculated quantitatively by Equation 5, where $i\in n,j\in m_{i},r_{j}\in R_{i}$

(5) $$\left\{\begin{array}{ccc} A_{ij} & = & \left\{r_{j}\right\}\\ A_{ij}^{\prime } & = & \left\{\text{min}\left(r_{j}\right),r_{j}\right\}\\ \vdots & \vdots & \vdots \\ A_{ij}^{n} & = & \left\{\text{min}\left(RandomP\left(A_{ij}^{n-1}\right)\right),A_{ij}^{n-1}\right\} \end{array}\right)$$

**Figure 3:**
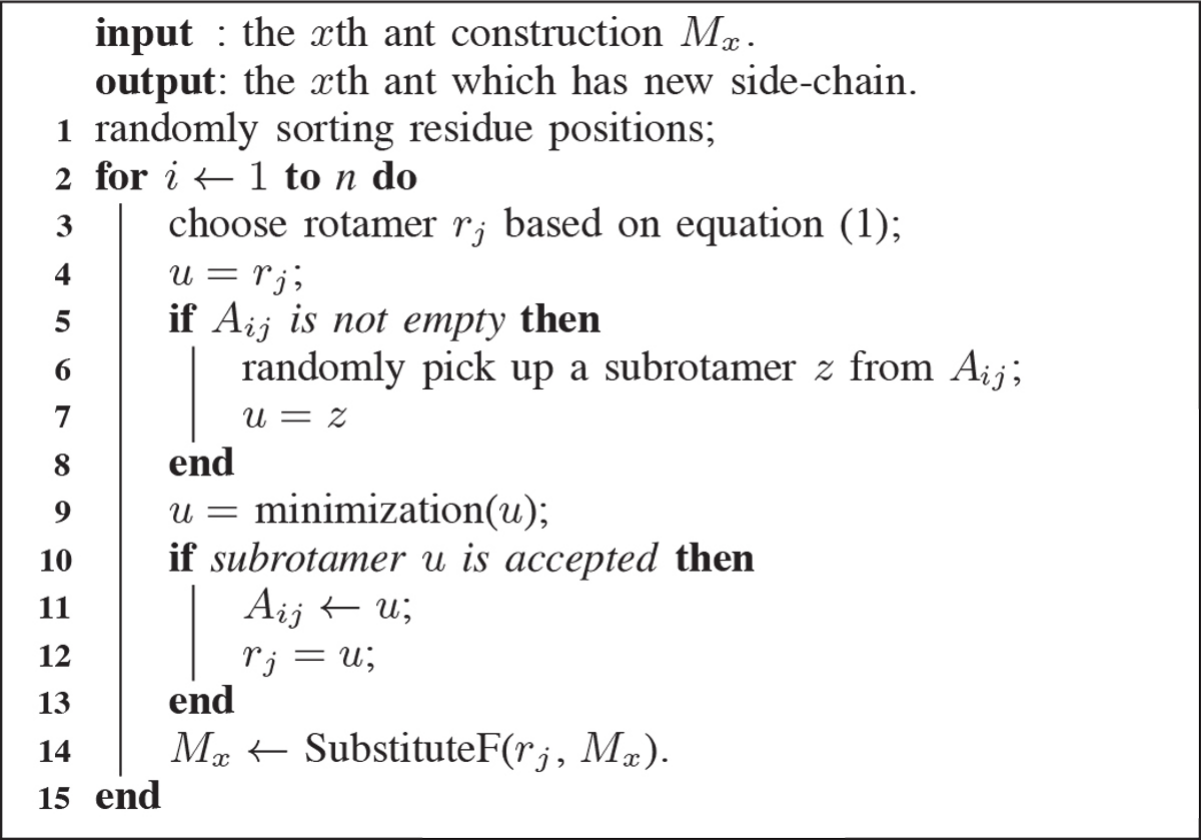
**Ant constructed side chains by minimising each placed rotamer**.

A detailed explanation of this equation is shown in Figure 3. An ant selects the rotamer *r_j _*for the *i^th ^*residue based on Equation 1, then find its subrotamers *A_ij _*as shown in step 5 in Figure 3, and randomly picks up a subrotamer from *A_ij _*to replace the primary rotamer at position *i*. The 9*^th ^*step attempts to optimise the subrotamer achieved by Rosetta. All minimisation algorithms in Rosetta choose a vector as the descent direction, determine a step along that vector, then choose a new direction and repeat [31]. We selected "dfpmin" as an exact line search for these steps. If this minimised subrotamer results in a drop in energy, it was kept and made into the residue *i*. Minimisation needs more time, so for researches with sufficient time who want to obtain more accurate results, this application would be a good choice.

## Results

The principal idea behind pacoPacker is to make the parallel ant colonies share only one pheromone matrix, which can combine different energies to guide each ant in constructing protein side-chain conformations. We tested pacoPacker by making comparisons with two popular side-chain modelling programs, CIS-RR and SCWRL4. CIS-RR combines a novel clash-detection guided iterative search (CIS) algorithm with continuous torsion space optimisation of rotamers (RR) [26]. SCWRL4 is an improved version of SCWRL3 [25] which uses the new rotamer library, more efficient search algorithms and a soft Vander Waals potential plus hydrogen bonding based scoring function [15]. All these predictors are based on discrete rotamers.

### Experimental settings

We performed all the tests on a computer cluster containing 20 nodes with 16-core 1.9 GHz AMD Opteron CPU per node under Linux 2.6.18 and GCC 4.1.2. CIS-RR and SCWRL4 were ran using their default settings to produce one prediction for each test instance. We ran pacoPacker, with eight ant colonies running in parallel, on the same test instances. As all these threads were synchronised to work out eight predictions and each is a nondeterministic approach, different numbers of decoys for each test instance were generated. The number of predictions for each test instance ranged from 2130 ([PDB:1CBN] 46 residues) to 4650 ([PDB:1B9O] 635 residues). We selected the highest accuracy rate of each test instance from pacoPacker to compare with CIS-RR and SCWRL4.

The benchmark instances were taken directly from other research, which contained 442 protein targets with lengths of 46 to 1184 amino acid residues [26,15]. Because [PDB:2QOL] cannot be predicted by CIS-RR and [PDB:1G8Q] is considered as a missing main chain atom by Rosetta, we excluded them from this benchmark. A fair evaluation is a difficult task, so we used two criteria to assess the accuracy of side chain packing. One was defined as the percentage of correctly predicted $X_{1}$ and $X_{12}$ angles within thresholds of 40° and 20° compared with the native structures. The second criterion was the root mean square deviation (RMSD) of the side-chain heavy atoms [34]. Both evaluation methodologies are adapted from third-party software [26,35], where they consider residues with symmetric terminal groups, or with a possibly flipped terminal group.

### Protein chain based evaluation performance

Firstly, we compared pacoPacker with CIS-RR and SCWRL4 in side-chain modelling. As shown in Table 3 for the accuracy improvement in terms of correct  X dihedral angles and RMSD, pacoPacker is comparable to the recently developed side-chain programs. As SCWRL4 showed relatively poor performance, so we only present a detailed comparison between pacoPacker and CIS-RR. Within 40*°*, the $X_{1}$ of the whole protein was improved by 2.31% with pacoPacker (87.19% by pacoPacker versus 84.88% by CIS-RR), and the *χ*12 was comparable (77.11% by pacoPacker versus 77.13% by CIS-RR). A similarly consistent trend was also seen for the accuracy rate of $X_{1}$ and $X_{12}$ within 20*°*. In case of the other metrics, pacoPacker is the best with its lowest RMSD.

**Table 3:** Comparison of pacoPacker, CIS-RR and SCWRL4 in the 442 structure set.

Method	$X_{1}\left(40^{{}^{\circ}}\right)$	$X_{1}\left(20^{{}^{\circ}}\right)$	$X_{12}\left(40^{{}^{\circ}}\right)$	$X_{12}\left(20^{{}^{\circ}}\right)$	RMSD (*Å*)
SCWRL4	82.80%	79.61%	74.98%	68.21%	2.07
CIS-RR	84.88%	82.07%	77.13%	70.13%	1.62
pacoPacker	87.19%	83.53%	77.11%	70.02%	1.60

**Figure 4:**
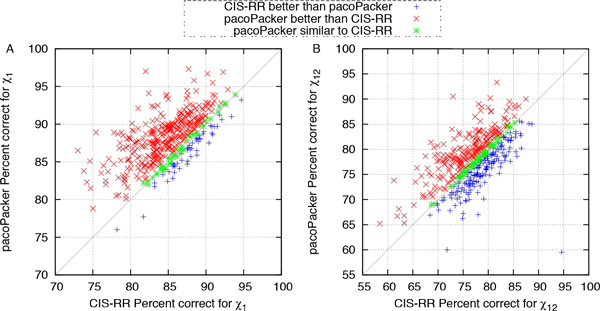
**Comparison of pacoPacker and CIS-RR for  X angles in the 442 structures set**. Each symbol corresponds to a single protein target. Red crosses (blue criss-crosses) denote pacoPacker yields better (worse) results. Targets marked by green asterisks mean pacoPacker is comparable with CIS-RR. All  X angles are within 40°.

**Figure 5:**
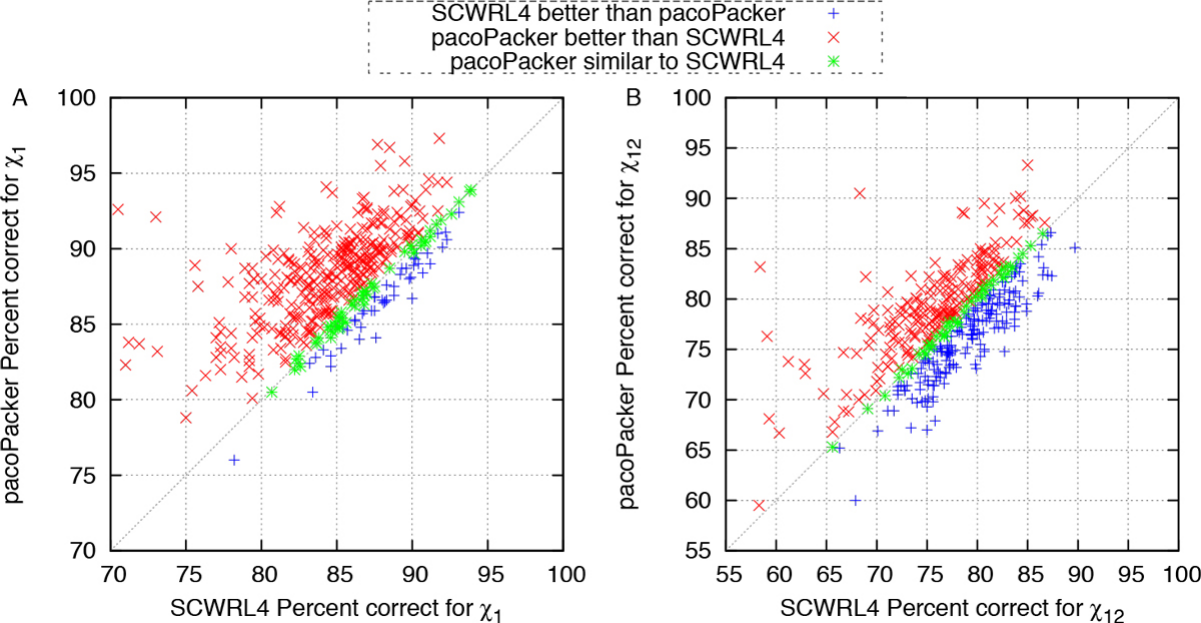
**Comparison of pacoPacker and SCWRL4 for  X angles in the 442 structures set. **Each symbol corresponds to a single protein target. Red crosses (blue criss-crosses) denote pacoPacker yields better (worse) results. Targets marked by green asterisks mean pacoPacker is comparable with SCWRL4. All  X angles are within 40°.

**Figure 6:**
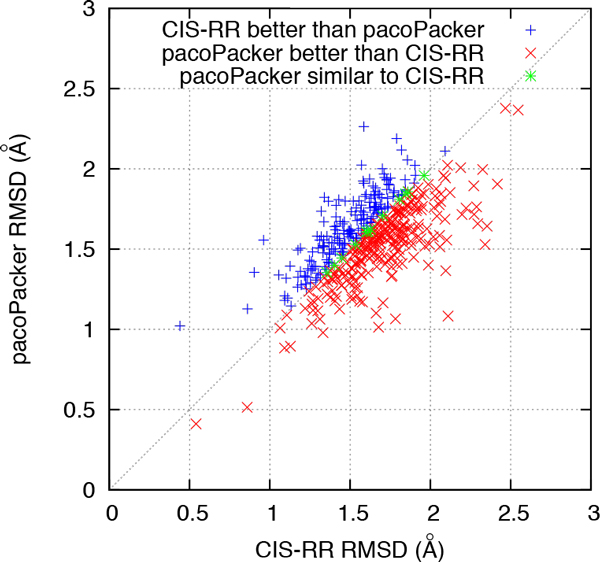
**Comparison of pacoPacker and CIS-RR for RMSD in the 442 structures set**. Each symbol corresponds to a single protein target. Red crosses (blue criss-crosses) denote pacoPacker yields better (worse) results. Targets marked by green asterisks mean pacoPacker is comparable with CIS-RR.

**Figure 7:**
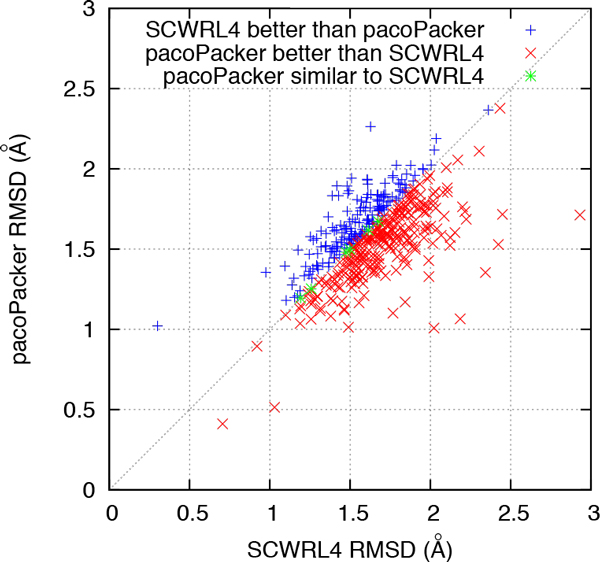
**Comparison of pacoPacker and SCWRL4 for RMSD in the 442 structures set**. Each symbol corresponds to a single protein target. Red crosses (blue criss-crosses) denote pacoPacker yields better (worse) results. Targets marked by green asterisks mean pacoPacker is comparable with SCWRL4.

We made further comparisons between the three predictors. In Figures 4 to 7, each symbol represents a single protein target, a red cross denotes a better pacoPacker yield and a blue criss-cross denotes a worse yield. Some differences between the two methods were less than 0.5% for the accuracy of  X dihedral angles and 0.005Å for RMSD, respectively. These are denoted by a green asterisk. As shown in Figures 4 and 6, when compared with CIS-RR, there were 342, 210 and 242 targets predicted by pacoPacker for $X_{1}$, $X_{12}$ and RMSD respectively, showing that it has the advantage over CIS-RR. Moreover, Figures 5 and 7 show that pacoPacker was better than SCWRL4 for 332, 211 and 267 targets for $X_{1}$, $X_{12}$ and RMSD respectively. These results clearly show that pacoPacker has a high reliability based on SHOP.

### Individual residues based evaluation performance

**Figure 8:**
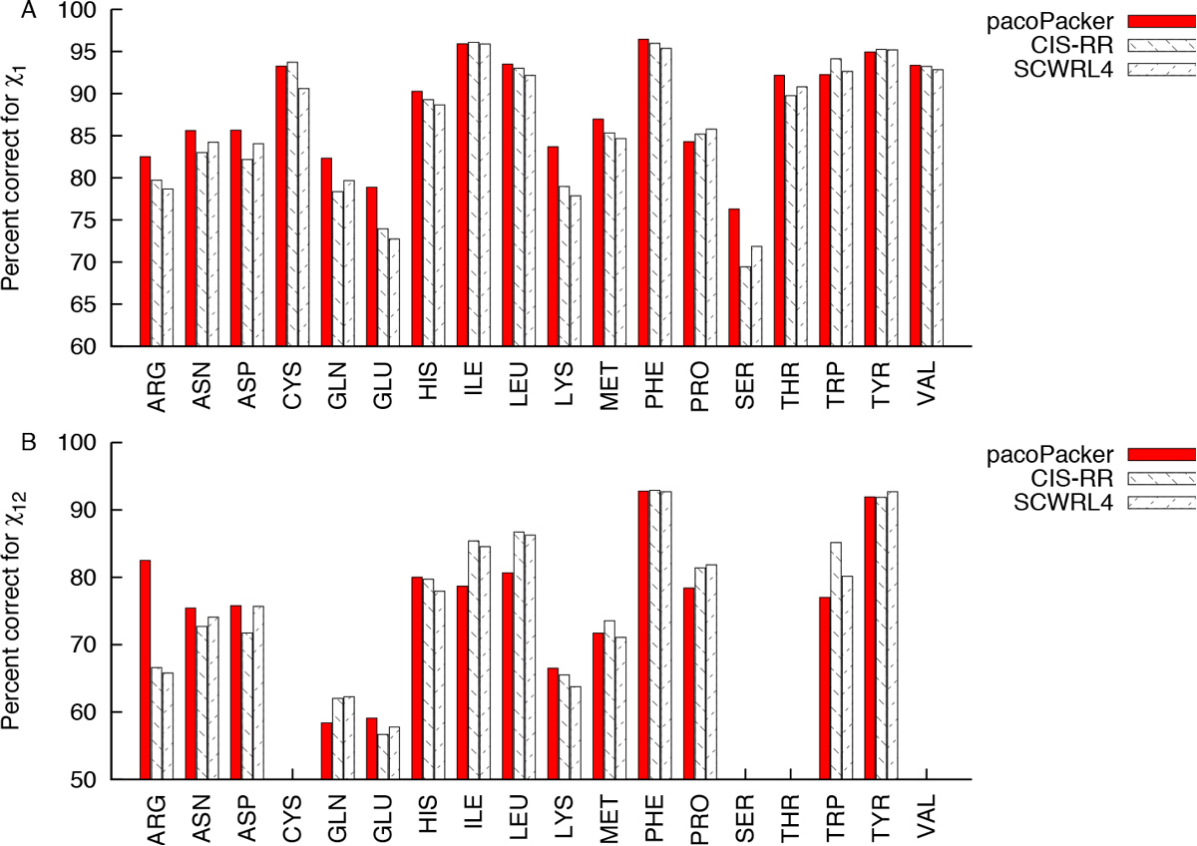
**Analysis of the performance of pacoPacker, CIS-RR and SCWRL4 on different amino acids**. (A) Percent correct within 40° for the $X_{1}$ angle. (B) Percent correct within 40° for the $X_{12}$ angle. The test data used the 442 proteins set. Red, back slash and forward slash indicate the test results of pacoPacker, CIS-RR and SCWRL4, respectively.

Next, we sought to evaluate how pacoPacker works on different types of amino acids. Figure 8 shows that pacoPacker improved the percent correct of both $X_{1}$ and $X_{12}$ dihedral angles. For $X_{1}$, excluding Ala and Gly, pacoPacker has 15 types of amino acids holding the top spot. In Glu, Lys and Ser, they had an average increase of more than 5%. PacoPacker made the greatest contribution to the accuracy of $X_{1}$. It also can be proven from the situation that pacoPacker made the greatest contribution to the accuracy of $X_{1}$ via its accurate prediction of Ser and Thr. The residues, which were predicted accurately, were predominantly aliphatic and aromatic residue types. For $X_{12}$, pacoPacker accounted for 6 types of amino acids in the lead, whilst CIS-RR accounded for 5 and SCWRL4 accounted for 3. Previous research has shown that for the short polar amino acids (Asp, Asn and Ser), CIS-RR shows lower performance, which could be due to the difference in scoring functions [26]. However, pacoPacker improves them both in $X_{1}$ and $X_{12}$, which has again shows the importance of combining different energies.

### Effects of rotamer minimisation

From the results presented in the previous two sections, we show the superiority of $X_{1}$ while the performance of $X_{2}$ is not strong. For example, when compare the number of red crosses on Figure 4(A) with Figure 4(B), pacoPacker has 342 best-performing proteins for $X_{1}$, which is more than the 210 best-performing proteins for $X_{12}$. In addition, Cys, Ser, Thr and Val only on wing $X_{1}$, clearly dominate the area of $X_{1}$. High quality $X_{1}$ is significant for side-chain prediction, because it is a foundation of residue. On the other side, there is still room for improvement of $X_{2}$, so we naturally optimised each rotamer as it was placed (rotamer minimization). An overview of how this method performs is given below.

**Figure 9:**
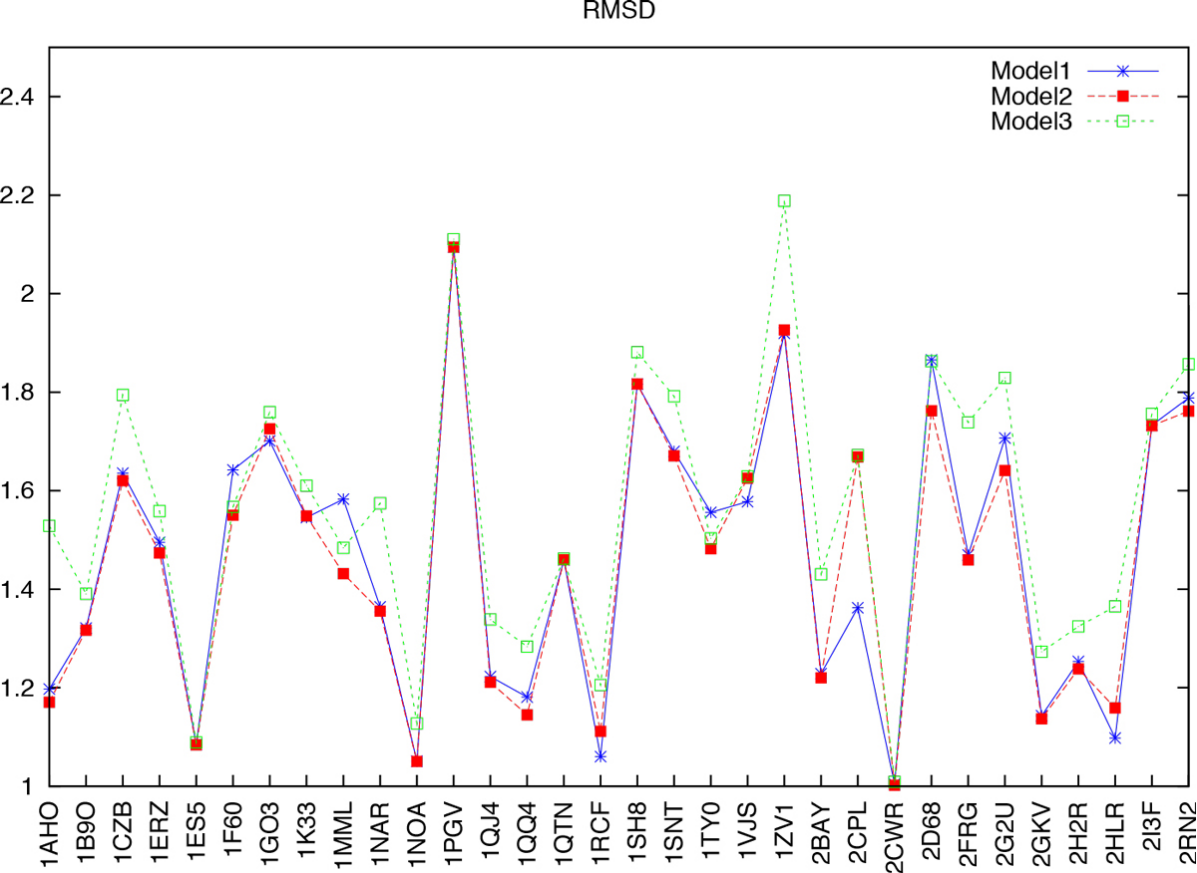
**Comparisons between the three models for RMSD**. Model 1 (blue asterisk) use a gradient minimisation on each rotamer when it is placed (this paper's method); model 2 (red solid box) packs using the same method as model 1, but then after the fact, runs a global minimisation on the side chains at all packable positions; and model3 (green box) with normal rotamers is optimised by global minimisation.

Figure 9 shows the effects of minimisation by comparing RMSD among three different models, and test instances is randomly from the benchmark as above. Model 1 (blue asterisk) uses gradient minimisation on each rotamer when it is placed (the method presented in this paper), model 2 (red solid box) packs the same way as model 1 but then runs a global minimisation on the side chains at all packable positions, and model 3 (green box) with normal rotamers is optimised by global minimisation only. Figure 9 shows that models 1 and 2 both decrease the RMSD compared with model 3, which means that our method can contribute to the quality of repacking. Most of time model 1 is comparable with model 2, so we can only use our method to gain optimisation as well as global minimisation. However, there were 18 proteins (data not shown), which had higher RMSD predicted by rotamer minimization. These can be classified into two groups: Those which already have high accuracies of $X_{1}$ and $X_{12}$ within 20*°*( with approximately 80% accuracy) and those which are large in size, including [PDB:2OTU] (976 residues), [PDB:1OK7] (739 residues), [PDB:1YTL] (631 residues), [PDB:2EPI] (388 residues). This means that structural integrity is important for proteins that are large in size, because rotamer minimisation cannot play a full role.

## Discussion

Under the inaccuracy/usefulness property hypothesis, SOP is not an ideal computational model for protein structure prediction [28]. This means that even if the corresponding SOP is completely solved, the SOP answer may not be correct, and in most cases it will not be perfect. PacoPacker proposes a novel hybrid parallel approach to repack protein side chains based on SHOP [28,30].

Table 4 shows the distribution of best conformations for each protein from pacoPacker on different threads. The best conformations are constructed on different threads, where each energy is very useful in some specific sense, but is inaccurate in a universal sense. Therefore, we need an approach based on MOP. For using MOP to solve protein structure prediction problems, the Pareto-based approach, which focuses on the dominance analysis of the solutions found by the search, will probably result in a large Pareto front with solutions where no single energy function can be dominant. PacoPacker is different as it does not construct a Pareto front, but collects the best solutions found by parallel search procedures directed by different energy functions. The SHOP strategy was proposed as a useful parallel ACO method [30]. Using SHOP, these multiple colonies of pacoPacker can exchange their search experiences asynchronously and co-evolve towards better solutions while each colony is guided by its own objective function and algorithm parameters [28]. In 442 structures test set, the close half targets of pacoPacker maintain optimum accuracy, unlike that in the other two programs. Why does the pacoPacker approach have a good performance?

**Table 4:** Best conformations of pacoPacker distributed on different threads.

ID	Thread0	Thread1	Thread2	Thread3	Thread4	Thread5	Thread6	Thread7
Quantity	29	31	35	29	33	39	107	139

Firstly, from the view of an individual colony, the pheromone matrix accumulates the search experience of ants, which describes which rotamer should be a priori considered as the choice for each residue. Such an experience bias is established by evaluating the conformations found by the previous generation of ants using the corresponding energy function. Then by sharing *T *, each colony can achieve different search experiences from other colonies asynchronously, and each colony is also directed by their own energy functions to co-evolve towards a better state. The process of sharing one *T *can accumulate the search experience of all parallel ant colonies and propagate the bias among them. As the pheromone matrix *T *provides an indeterministic bias for all the running colonies, it may be easier to find better solutions.

For example, [PDB:2FLU] was one of the most accurate predictions from paco-Packer with a RMSD of 0.98, while the second most accurate prediction was 1.33 from CIS-RR. The best conformation appeared in the 27*^th ^*generation of thread 8, which ends on this generation. The other threads ended incrementally after the 29*^th ^*generation. In this situation, almost all threads stop at the same time, which gives pheromone matrix *T *enough time to learn experiences fairly from different threads. There were some poor solutions, such as [PDB:1WVH] where the RMSD was increased by 1.23 with pacoPacker. In this case, the best conformation of pacoPacker was structured by thread 6 on the 40*^th ^*generation, and other threads stopped after 25*^th ^*generation. This may be because some threads accomplish too early so that the pheromone matrix *T *learns search experiences with bias, which may be solved with more time. From a user perspective, we summarise when pacoPacker performs well in Table 5. This shows that the proportion of proteins repacked increased as the sequence length decreased. Therefore pacoPacker can provide the highest accuracy for packing side chains when the sequence length is lower than 400 amino acids.

**Table 5:** The proportion of proteins repacked by pacoPacker with lower RMSD compared with other predictors.

Sequence Length	Number	CW CIS-RR	CW SCWRL4	CW both
*>*500	53	28.3%	30.2%	13.2%
500*~*400	34	41.2%	38.2%	20.6%
400*~*300	62	56.5%	62.9%	41.9%
300*~*200	108	59.3%	66.7%	51.9%
200*~*100	139	63.3%	67.6%	51.1%
*<*100	46	76.1%	76.1%	63.0%

The first column denotes the range of sequence length; the second column records the number of proteins; the remaining columns show the proportion of proteins packed side chains by pacoPacker with smaller RMSD than CIS-RR or SCWRL4 alone, or combined.

## Conclusions

In summary, pacoPacker makes each heuristic search work with its own energy function and they complement each other in a qualitative way. Different energy functions train search trajectories to obtain different search intelligences. Our parallel strategy diffuses the intelligence to all the parallel searches by SHOP, so that all ant colonies can share their accumulated hybridised intelligence. Such co-evolvement guided by multiple objective functions simultaneously has an impact on the nature folding procedure of native proteins [28]. The prediction accuracy of packing side chains was improved for most of the proteins, which proves that pacoPacker has feasibility and practical value, but at a cost of increased CPU time. However, an important reason for using pacoPacker is that it does not need training and tuning of the energy function parameters before the predictor can work.

## Competing interests

The authors declare that they have no competing interests.

## Authors' contributions

Q Lü designed and developed the pacoPacker framework. LJ Quan implemented and improved pacoPacker. LJ Quan, HO Li and HJ Wu performed the experiments. LJ Quan and XX Xia drafted the manuscript. All of the authors read and approved the manuscript.

## Declarations

This study was supported by a grant from the National Natural Science Foundation of China (No. 61170125).

This article has been published as part of *BMC Bioinformatics *Volume 15 Supplement 12, 2014: Selected articles from the IEEE International Conference on Bioinformatics and Biomedicine (BIBM 2013): Bioinformatics. The full contents of the supplement are available online at http://www.biomedcentral.com/bmcbioinformatics/supplements/15/S12.
